# ELABELA Targets Mitochondria to Modulate Heart Development

**DOI:** 10.1002/advs.202506525

**Published:** 2026-04-07

**Authors:** Jian Wang, Qingjie Wang, Zhikang Xu, Shuang Zhou, Yue Zhou, Junjie Yang, Lingfeng Tong, Zhuo Meng, Mei Yang, Wen Zhao, Tie Yang, Hualin Wang, Jun Zhang, Rubin Tan, Lei Wang, Yuqiang Huang, Bin Zhou, Sun Chen, Bing Zhang, Jinxiang Yuan, Jianyuan Zhao, Alex F. Chen, Kun Sun

**Affiliations:** ^1^ Department of Pediatric Cardiology Xinhua Hospital Affiliated to Shanghai Jiaotong University School of Medicine Shanghai China; ^2^ Institute for Developmental and Regenerative Cardiovascular Medicine Xinhua Hospital Affiliated to Shanghai Jiaotong University School of Medicine Shanghai China; ^3^ Engineering Research Center of Medical Devices For Congenital Heart Disease Ministry of Education Xinhua Hospital Shanghai Jiao Tong University School of Medicine Shanghai China; ^4^ Department of Cardiology Xinhua Hospital Affiliated to Shanghai Jiaotong University School of Medicine Shanghai China; ^5^ Ministry of Education‐Shanghai Key Laboratory of Children's Environmental Health Xinhua Hospital Shanghai Jiao Tong University School of Medicine Shanghai China; ^6^ Department of Physiology Basic Medical School Southwest Medical University Luzhou Sichuan China; ^7^ Diagnosis and Treatment Center For In Utero Pediatric Diseases Xinhua Hospital Affiliated to Shanghai Jiao Tong University School of Medicine Shanghai China; ^8^ Linyi Maternal and Child Health Care Hospital Linyi Shandong China; ^9^ New Cornerstone Science Laboratory State Key Laboratory of Cell Biology Shanghai Institute of Biochemistry and Cell Biology Center For Excellence in Molecular Cell Science Chinese Academy of Sciences University of Chinese Academy of Sciences Shanghai China; ^10^ Lin He's Academician Workstation of New Medicine and Clinical Translation Jining Medical University Jining Shandong China

**Keywords:** BCL2/BAX, congenital heart disease, ELABELA, mitochondria

## Abstract

Congenital heart disease (CHD) is a leading cause of neonatal morbidity and mortality, whose underlying pathogenesis remains largely unclear, and lacks reliable biomarkers or therapeutic targets for early detection and treatment during pregnancy. In this study, we investigated the role of endogenous peptide ELABELA (ELA) in fetal CHD. Our findings reveal that ELA levels are significantly reduced in human fetal cardiac tissues with CHD. In mouse models, ELA deletion in cardiac progenitor cells disrupted mitochondrial function, directly contributing to cardiac malformations. Mechanistically, ELA deficiency caused mitochondrial swelling by inhibiting the APJ‐AKT‐BCL2/BAX signaling pathway. Notably, exogenous ELA administration reduced both CHD severity and incidence in mice. Furthermore, plasma ELA levels were markedly down‐regulated in human pregnancies with fetal CHD. These findings establish ELA as a crucial regulator of cardiac development and highlight its potential as both a biomarker and therapeutic target for the prevention and management of fetal CHD during gestation.

## Graphical abstract

The role of peptide ELABELA (ELA) in cardiomyocyte apoptosis and congenital heart disease (CHD) is unclear. ELA deficiency caused cardiomyocyte apoptosis and CHD. A novel ELA‐APJ‐AKT‐BCL2/BAX axis in regulating mitochondrial function and contributing to CHD pathogenesis was established. Exogenous ELA peptide administration to pregnant mice reduced the incidence and severity of cardiac anomalies as well as the lethality rates of embryos. ELA peptide was a potential biomarker for human CHD during pregnancy. These novel findings show that mitochondrial swelling associated with congenital heart disease. Targeting ELA‐APJ‐AKT‐BCL2/BAX axis represents a new strategy to screen and prevent CHD.

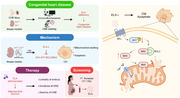

## 1 Introduction

Congenital heart disease (CHD) refers to structural or functional abnormalities of the heart that are present at birth, arising from improper development of the heart or major blood vessels during fetal development. As one of the most common congenital malformations, CHD affects approximately 1% of live births [1, 2]. Among the various cell types in the heart, cardiomyocytes (CMs) are the main cell group of heart responsible for cardiac contraction [3, 4]. During the development of heart, the balance between proliferation and apoptosis of CMs is critical for normal heart growth [5, 6], the disturbance of this balance could play an important role during the onset of CHD [7, 8]. As exemplified by ventricular septal defect (VSD), the most common subtype of CHD, aberrant CMs apoptosis could impair proper septal formation [9, 10].

Growing evidence highlights the pivotal role of peptides in cardiogenesis, underscoring their essential involvement in signal transduction pathways [11, 12]. One such peptide, ELABELA (ELA), encoded by the APELA gene, originally annotated as non‐coding DNA, is preserved across evolutionary boundaries [13]. The APELA gene was predicted to express a 54‐amino acid protein comprising a 32‐amino‐acid peptide (ELA‐32, mature form without the signal peptide). Although there are several other subtypes of ELA peptides, including ELA‐21, ELA‐14, and ELA‐11 [14, 15], the 32‐amino‐acid mature ELA peptide shows over 80% sequence identity across zebrafish, mouse, and human [16]. In addition, ELA‐32 is the longest isoform, containing the sequences of several other subtypes. Therefore, ELA‐32 was selected for further investigation in this study.

Previous studies have found that reduced expression of ELA in vertebrates has been linked to various developmental abnormalities, including defective endoderm differentiation [17], impaired cell migration during gastrulation that leads to vascular defects [18], and congenital heart malformations resulting from disrupted migration of cardiac progenitor cells [13]. ELA deficiency in mice is associated with embryonic lethality and cardiovascular malformations, including defective heart looping and impaired vascular remodeling [16, 19]. Despite these findings, the precise role of ELA in human embryonic heart development and its contribution to the pathogenesis of cardiac malformations remain insufficiently understood and warrant further investigation.

This study emphasizes the pivotal role of the ELA peptide in cardiac development. Our research demonstrates that ELA is dynamically expressed in the developing human embryonic heart, yet its levels are significantly diminished in the cardiac tissues of fetuses with CHD. In mouse models, ELA deficiency in cardiomyocytes (CMs) during early cardiac morphogenesis leads to outflow tract defects. Mechanistically, our findings revealed that ELA deficiency enhanced mitochondrial permeability and disrupted mitochondrial function in CMs by inhibiting the activation of the APJ‐AKT‐BCL2/BAX axis, ultimately resulting in apoptosis. Notably, administering exogenous ELA during pregnancy markedly improves the survival rates and reduces the incidence of cardiac anomalies in ELA‐deficient offspring. Additionally, we identified a strong correlation between reduced maternal plasma ELA levels and the occurrence of CHD in offspring. These data suggest that ELA may serve as a promising therapeutic target and a maternal biomarker for fetal CHD (fCHD) during gestation.

## 2 Results

### 2.1 ELA Expression in Human Cardiac Tissues

ELA deficiency has been linked to cardiac malformations in animal models [19]. However, its specific role in human heart development has not been addressed. To elucidate the contribution of ELA to human cardiac development, we first investigated its expression patterns during embryogenesis.

Human organogenesis spans Carnegie Stages 9–23 (embryonic day 20, E20 to ∼8 weeks), with Stage 23 marking the embryonic terminus after which development enters the fetal phase persisting through the second and third trimesters. The intricate processes of heart looping, chamber formation, and the separation of the ventricles, atria, and outflow tract (OFT) take place mainly between embryonic day 22 and day 42, spanning across six sequential Carnegie stages (CS11‐CS16) [20, 21]. To capture this critical window of heart development, we collected human embryos from medical terminations from stages CS11 to CS16, thereby covering the pivotal phase of cardiac formation [20, 22].

**FIGURE 1:**
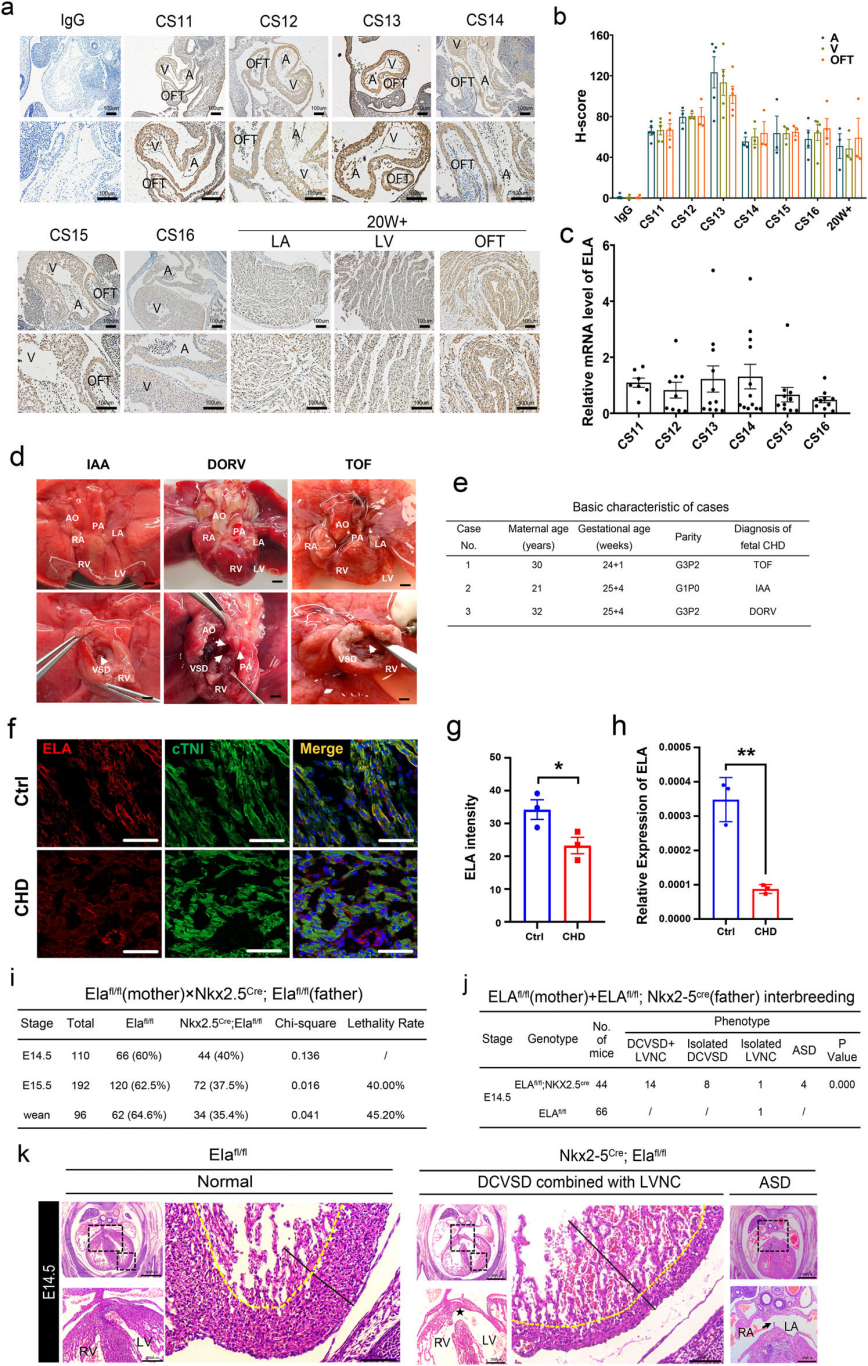
**ELA deficiency leads to cardiac outflow tract anomalies**. (a) Immunohistochemistry of ELA in human embryonic and fetal hearts during Carnegie stage (CS)11‐CS16. Scale bars, 100 µm. (b) The graph of *H* Scores of (**a**) (mean ± SEM). (IgG, n = 6; embryonic heart, n = 5 at CS11 and CS13; n = 3 at CS12, CS14, CS15; n = 4 at CS16; fetal heart, n = 3). (c) qPCR of the relative mRNA level of ELA in human embryonic hearts during CS11‐CS16. (n = 7 at CS11; n = 9 at CS12; n = 12 at CS13 and CS14; n = 11 at CS15; n = 10 at CS16). (d) Overview of fetal hearts with IAA, DORV, and TOF respectively. Scale bars, 1 cm. (e) Basic characteristic of samples in (d). (f) Immunofluorescence detects ELA decreases in heart tissue from fetus with CHD and matched controls. Scale bars, 75um. (g) Intensity of fluorescence in (f). (h),qPCR validation of ELA expression in fetal CHD (n = 3) and matched controls (n = 3). (i), Distribution of genotypes obtained from intercrossing ELA^fl/fl^ female to ELA^fl/fl^; Nkx2‐5^cre^ male mice. (j), The phenotypic spectrum of ELA^Nkx^ mice. (k), Hematoxylin and eosin (H&E) staining was performed on transverse sections at E14.5 of ELA^Nkx^ vs. control mice. Asterisks indicate VSD; Black arrowhead denote ASD. RV, right ventricle; LV, left ventricle; RA, right atrium; LA, left atrium. Scale bars, 500, 250 and 100 µm respectively. For all, ^*^ *p*<0.05, ^**^ *p*<0.01. For (b) and (d), data are represented as mean ± SEM; Two‐tailed t test was applied for (g) and (h). For (e), (i), and (j), Chi‐square test was applied.

We evaluated the dynamic expression of ELA across these developmental stages using immunohistochemistry and quantitative real‐time PCR (qPCR) assays. Our analysis revealed that ELA was consistently expressed in the developing human embryonic heart, with its localization restricted to the OFT, atrium (A), and ventricle (V) throughout stages CS11 to CS16 (Figure 1a–c). Further characterized of ELA expression patterns, we collected human fetal hearts at gestational weeks 24–‐26, a developmental stage with specimens exhibiting complete anatomical maturation including established four‐chamber architecture and functional coronary circulation. In fetal tissues affected by CHD with OFT malformation (including interruption of aortic arch, IAA; double outlet of right ventricle, DORV; tetralogy of fallot, TOF), we detected a significant reduction in ELA expression within the myocardium (Figure 1d–h). These findings illustrate the critical role of ELA in human heart development and suggest that its deficiency is associated with fCHD.

### 2.2 Cardiac‐Specific Deletion of *Ela* in Early Heart Development

To better understand ELA's role in heart development, we examined heart tissue from mouse embryos and noted that the ELA expression pattern strongly resembles that seen in human and mouse embryonic hearts (Figure 1a,c; Figure S1a). We subsequently generated an Ela‐DreER‐IRES‐GFP knock‐in mouse model (Figure S1b), where GFP was expressed under the control of the ELA promoter, providing a clear and reliable marker for ELA expression. Double immunostaining for ELA and Nkx2‐5 revealed that ELA was predominantly expressed in Nkx2‐5 positive cardiac progenitor cells in the developing E8.5 heart (Figure S1c).

Previous studies have shown that global knockout of ELA leads to embryonic lethality in mice, characterized by abnormal cardiac tube looping [13, 19]. However, the specific role of ELA in the formation of cardiac malformations remained unclear, and so did the underlying mechanisms. To better investigate the specific role of ELA early in heart development, we implemented a conditional knockout (CKO) strategy by utilizing Nkx2‐5‐Cre mice to specifically delete ELA in cardiac progenitor cells. We crossed homozygous ELA^flox/flox^ mice with Nkx2‐5‐Cre mice and subsequently obtained ELA^flox/flox^; Nkx2‐5‐Cre (ELA^Nkx^, ELA CKO) embryos (Figure S1d–f). We collected and analyzed the embryos from embryonic day 9.5 (E9.5) to the weaning stage, and the results showed a significant decrease in the survival rate of homozygous offspring at E15.5 (Figure 1i; Figure S2a).

Notably, ELA^Nkx^ mice exhibited pericardium edema at E9.5 and E10.5 (Figure S2a,b). At E14.5, ELA^Nkx^ mutants had multiple cardiac defects, including doubly committed subarterial ventricular septal defect (DCVSD) accompanied with or without left ventricular non‐compaction (LVNC) and atrial septal defect (ASD) (Figure 1j–k). These findings demonstrate that cardiac deletion of ELA led to poorly developed myocardium, highlighting the crucial role of ELA in regulating cardiac development.

### 2.3 Gene Expression Alterations in ELA Deficiency CMs

**FIGURE 2:**
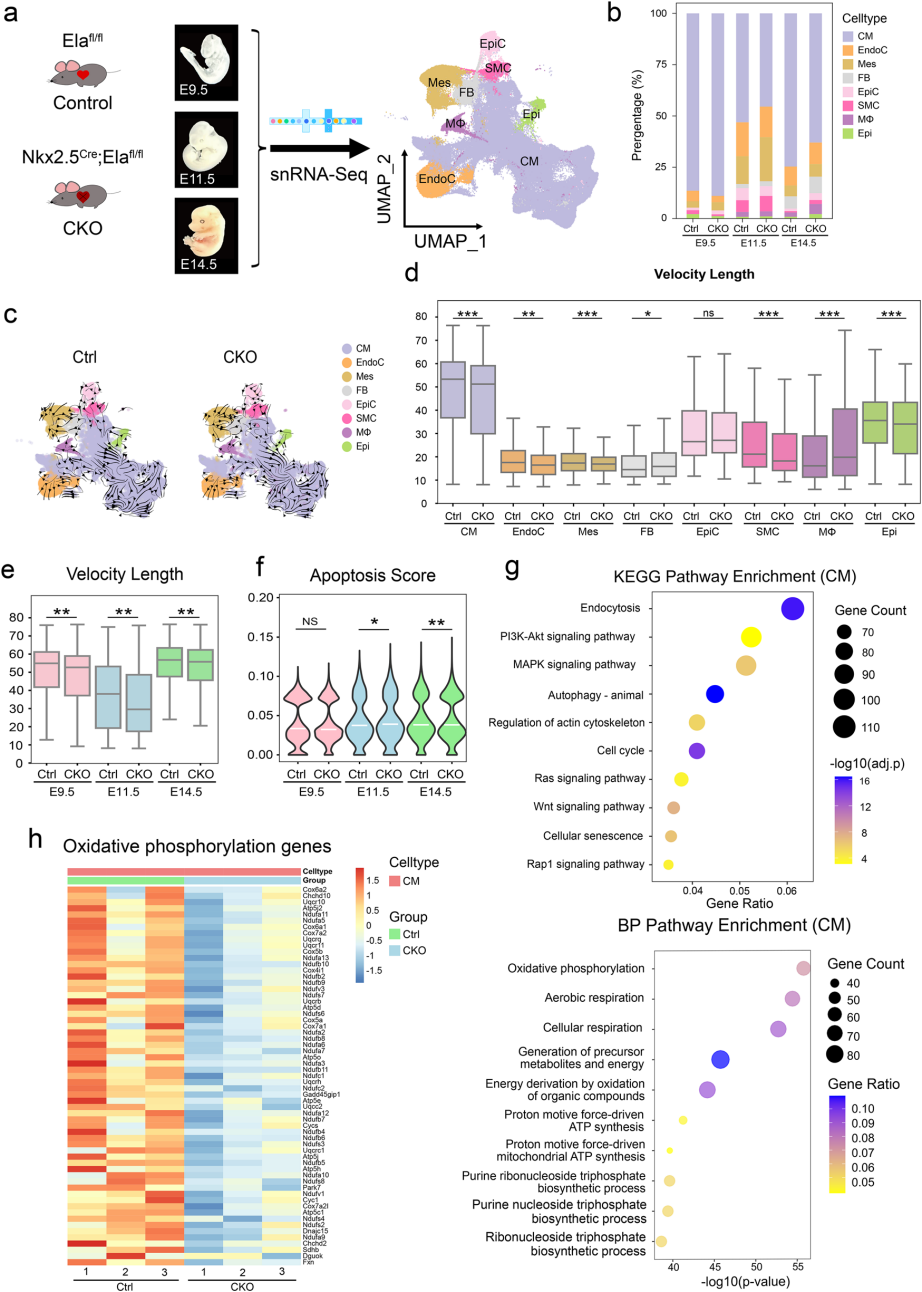
Single‐cell analysis reveals that ELA disruption alters developmental trajectories and differential gene expression profiles in CM. (a), Left, schematic of study. Right, UMAP clustering of single cells from control and ELA conditinal knock out (CKO) cardiac tissue (E9.5, E11.5, and E14.5). Cells are colored by type. (b), The percentage of each cell type is counted at E9.5, E11.5, and E14.5. (c), The RNA velocity plots of control and CKO samples. (d), Length of the velocity vectors across different cell types. (e), Length of the velocity vectors in cardiomyocytes at different stages. (f), The violin plot shows the apoptosis score in cardiomyocyte between control and CKO groups. (g), Downregulated pathways that are enriched in E11.5 CKO embryonic cardiomyocytes compared with control. Upper, KEGG pathway enrichment, Lower, BP pathway enrichment. (h), Heatmap showing the average expression for the representative differentially expressed genes in (g). For all, ^*^ *p* < 0,05; ^**^ *p* < 0,01; ^***^ *p* < 0,001. Welch's t test was applied for (d–f).

To gain a deeper understanding of the changes occurring in specific cardiac developmental processes within ELA^Nkx^ (ELA CKO) embryonic hearts, we performed single‐cell RNA sequencing (scRNA‐seq) at three critical developmental stages (E9.5, E11.5, and E14.5) on embryonic hearts (Figure 2a), which helped capture dynamic gene expression profiles across these three critical time points. By employing an unsupervised analysis strategy [23], we clustered the single‐cell transcriptomes and successfully identified distinct cell populations based on well‐established cell type‐specific markers and previously documented lineage information. These identified clusters include CMs, fibroblasts (FB), endothelial cells (EndoC), epicardial cells (EpiC), mesenchymal cells (Mes), smooth muscle cells (SMC), macrophages (MФ), and epithelial cells (Epi) (Figure 2a; Figure S3a,b and Table S1).

Although the overall distribution of cell types in ELA^Nkx^ embryonic hearts was not significantly different from the control group, notable changes were observed in the proportions of various cell populations. As cardiac development progresses, we observed a continuous decline in the number of CMs, coupled with a corresponding increase in Mes after ELA deletion, highlighting the dynamic shifts in cellular composition among E9.5, E11.5, and E14.5 (Figure 2b).

To further explore the impact of ELA on cell fate determination, we conducted RNA velocity analysis, a method that predicts the direction and rate of cellular state transitions by comparing the relative levels of spliced and unspliced transcripts [24]. Velocity analysis showed that the arrows in ELA CKO group were thinner and fewer, compared to the control group. Moreover, the streamline plot of UMAP showed that the difference of developmental disparity at E11.5 was greatest (Figure 2c; Figure S3c). The velocity length highlighted the dynamic state changes in CM (Figure 2d,e). These findings suggested that the absence of ELA had the greatest impact on E11.5 CMs. The apoptosis score was significantly elevated in the CMs of ELA CKO embryonic heart compared to the control at E11.5 and E14.5, indicating that CMs were undergoing cell death in response to ELA deficiency (Figure 2f). We next performed gene set enrichment analysis, which revealed a significant downregulation of genes involved in oxidative phosphorylation, aerobic respiration, and related mitochondrial metabolic pathways. Concurrently, key signaling pathways including PI3K‐Akt, MAPK, and WNT were also suppressed, as evidenced by the significant downregulation of their constituent genes. These transcriptomic findings indicate that ELA deficiency in cardiomyocytes leads to the coordinated repression of mitochondrial metabolic programs and critical pro‐survival signaling cascades (Figure 2g,h).

### 2.4 Mitochondrial Swelling and Impaired Function in ELA Deficiency CMs

**FIGURE 3:**
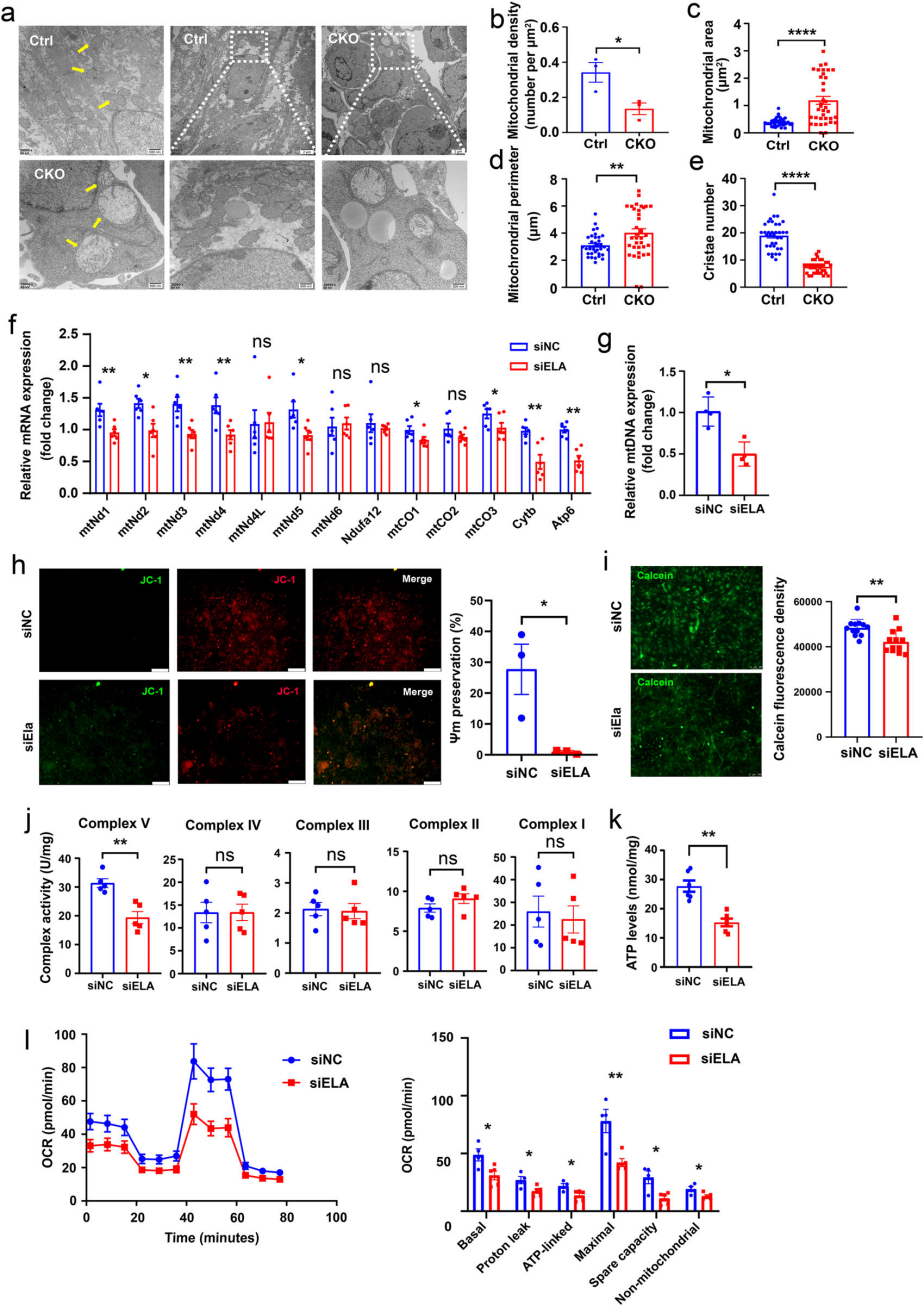
**ELA deficiency induced mitochondrial swelling and dysfunction in CM**. (a), Transmission electron microscope of E11.5 heart of control and ELA CKO mouse embryos (n = 3). Left, Swelling mitochondria, Scale bars, 500 nm for 2500x. Middle and Right, lipid accumulation. Scale bars 2um for 7000x, 500 nm for 2500x. (b–e), Quantification of (b) mitochondrial density, (c) mitochondrial area, (d) mitochondrial perimeter, (e) cristae number in control and ELA CKO mouse embryonic hearts (n = 3 for b, n = 36 for c, d, and e). (f), Mitochondrial structural and functional gene expression levels (n = 6). (g), Mitochondrial DNA levels detected by qPCR (n = 4). (h–m), Analysis of mitochondrial function in primary rat cardiomyocytes after treated with siRNA, including (h) mitochondrial membrane potential, (i) mitochondrial permeability, (j) activity of mitochondrial respiratory complex I–V, (k) ATP levels (l) oxygen consumption rate (OCR) (n = 3 for h, n = 12 for i, n = 5 for j, n = 6 for k, n = 5 for j‐l). Scale bars (h), 75 µm. For j, Scale bars (i), 100 µm. OCR were normalized to total protein content. For all, ^*^ *p*<0.05, ^**^ *p*<0.01, ^***^ *p*<0.001, ^****^ *p*<0.0001. Data are represented as mean ± SEM. Two‐tailed t test was applied for b‐l.

Based on multi‐time point single‐cell sequencing data, ELA deficiency caused the most pronounced changes in CMs at the E11.5 developmental stage. To further investigate these cellular and structural alterations, heart samples from E11.5 ELA^Nkx^ and control embryos were collected and analyzed using transmission electron microscopy (TEM) following standardized procedures established in previous studies [25, 26, 27]. The TEM analysis revealed that, compared to control embryos, CMs in OFT of E11.5 ELA CKO embryos exhibited significantly swollen mitochondria (Figure 3a). In ELA‐knockout embryonic cardiomyocytes, the mitochondria exhibited significant increases in perimeter, width, and area, alongside a marked reduction in both the density and number of cristae. Mitochondrial length was unchanged. Furthermore, lipid droplets displayed a significant expansion in size, as indicated by increases in their perimeter, area, and diameter (Figure 3b–e; Figure S4a–e).

Following this, neonatal primary CMs (NCMs) were isolated, and siRNA was utilized to suppress ELA expression (Figure S4f). The analysis demonstrated that ELA suppression led to a decrease in the expression of most mitochondrial morphological and functional genes (Figure 3f) alone with a substantial reduction in mitochondrial DNA content (Figure 3g).

Research demonstrated that mitochondria played an important role in maintaining CMs energy metabolism during heart development [28, 29, 30]. To further illuminate the mechanism behind the effect of ELA on mitochondrial function, we evaluated mitochondrial key biological processes, including mitochondrial biogenesis, dynamics, and autophagy [31].

We first analyzed the mitochondrial supercomplex formation by Blue native page analysis [31] and found no differences between control and CKO groups (Figure S5a). We then analyzed the expression of the key factor for mitochondrial fusion (mitofusins, MFN1 and MFN2, OPA1), mitochondrial fission (dynamin‐related protein 1, DRP1, p‐DRP1^S637^, p‐DRP1^S616^), mitochondrial proliferation (peroxisome proliferator‐activated receptor gamma coactivator‐1, PGC1α), and mitophagy (Prohibitin, PINK1, and LC3B). MFN1/2, OPA1, TFAM, PGC‐1α, and p‐DRP1^S616^ significantly decreased, while others showed no significant changes between control and CKO groups (Figure S5b).

Therefore, we assumed that ELA impaired mitochondrial function through impact on mitochondrial permeability and mitochondrial membrane potential. We investigated these two indices and found a significant decrease in ELA‐knockdown NCMs (Figure 3h,i). In addition, we found a marked elevated mitochondrial ROS levels (Figure S4g). Due to the former changes of mitochondrial function, we further assessed the complex I to V of mitochondria. Surprisedly, the Complex V, which is also known as ATP synthase, showed a dramatically decrease (Figure 3j). Therefore, we detected ATP content and also found a significant decrease in ELA‐knockdown NCMs (Figure 3k). Seahorse analysis revealed a significant reduction in oxygen consumption rate (OCR) in ELA‐knockdown NCMs, while the extracellular acidification Rate (ECAR) increased significantly (Figure S4i).

Together, these findings demonstrate that the disruption of ELA led to mitochondrial dysfunction, highlighting its critical role in ensuring cellular energy balance during embryonic heart development.

### 2.5 ELA Promotes Mitochondrial Function

Previous researches reported various mechanism of the regulation of mitochondrial permeability and mitochondrial membrane potential. Among these factors, BAX played a dominant role. Besides, previous studies also identified APJ‐AKT axis as a key downstream pathway of ELA [32].

**FIGURE 4:**
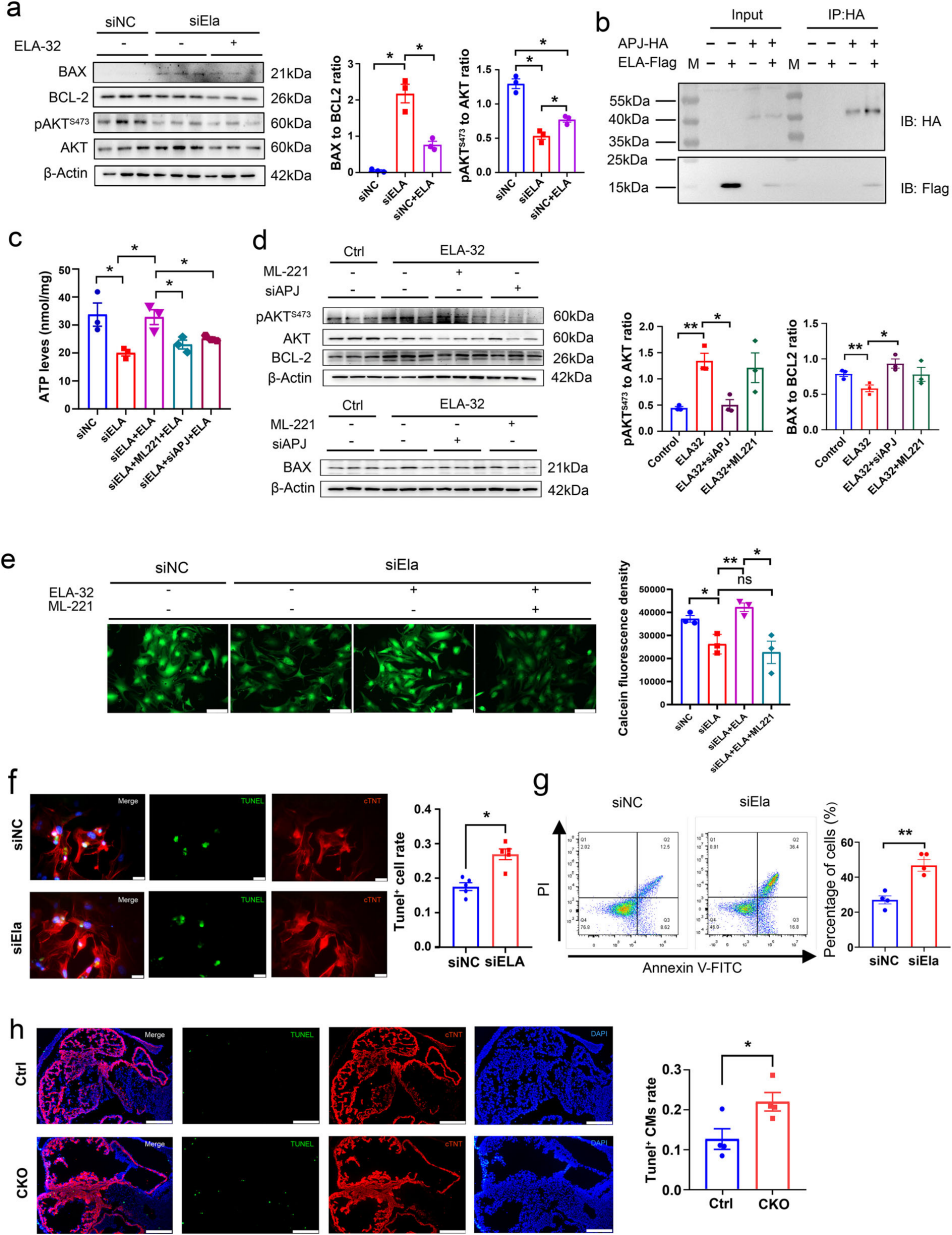
**ELA‐deficiency promotes cardiomyocyte apoptosis through AKT phosphorylation‐dependent downregulation of BAX**. (a), The expression of BAX, BCL‐2, pAKT^S473^, AKT in mouse NCMs after ELA knockdown by siRNA and the treatment of ELA‐32 were measured by WB. (b), The combination of ELA and APJ was detected by CoIP. ELA‐Flag and APJ‐HA were overexpressed together, and a vector was transfected as a control. Immunoprecipitation of APJ‐HA co‐pulled down ELA‐Flag. (c), The ATP levels in rat NCMs after knockdown of ELA with siRNA and inhibition of APJ with either ML‐221 or siAPJ (n = 3). (d), The expression of BAX, BCL‐2, pAKT^S473^, AKT in mouse NCM after ELA, ML‐221 treatment, and APJ knockdown by siRNA (n = 3). (e) mitochondrial permeability in mouse NCMs after treated with siELA, ELA‐32, and ML‐221 (f–g), The apoptosis of mouse NCMs after transfecting siRNA by immunofluorescence (f) and flow cytometry (g). Scale bars, 25 µm. (h), The tunnel staining of mouse embryonic heart tissues at E11.5. Scale bars, 100 µm. For all, ^*^ *p*<0.05, ^**^ *p*<0.01. Data are represented as mean ± SEM. Two‐tailed t test was applied for (c), (e), (f), (g), and (h).

Therefore, we evaluated the related factors changes after ELA knockdown in NCMs. The results showed a significant reduction in AKT phosphorylation alongside a marked upregulation of BAX protein expression and a significant downregulation of AKT phosphorylation (Figure 4a). However, after the treatment of ELA‐32, the levels of BAX and AKT phosphorylation were rescued (Figure 4a). As APJ was the important receptor, which mediated ELA and APJ‐AKT axis. First, we verified the combination of ELA and APJ by coimmunoprecipitation (CoIP) (Figure 4b). To delve deeper into the underlying mechanism, we applied the APJ inhibitor ML‐221 (10 µm) and performed APJ knockdown with siAPJ following ELA‐32 treatment. The ATP levels were decreased after pharmacological and genetical inhibition of APJ (Figure 4c; Figure S4h). Interestingly, a significant decrease was detected in AKT phosphorylation, and a significant increase in BAX expression was detected following the inhibitor treatment (Figure 4d). The mitochondrial function was further evaluated. A significant increase of mitochondrial permeability was showed following the inhibitor treatment (Figure 4e). The changes of mitochondrial permeability led to cell apoptosis. Therefore, the CM apoptosis was further evaluated. ELA knockdown significantly increased apoptosis in NMCMs, confirmed by immunofluorescence and flow cytometry (Figure 4f,g). Similarly, immunofluorescence revealed a significant increase in apoptotic CMs in E11.5 ELA^Nkx^ embryos (Figure 4h).

Together, these findings indicate that ELA deficiency induces CMs apoptosis by modulating BAX expression through AKT phosphorylation, ultimately influencing mitochondrial permeability.

### 2.6 Maternal Circulating ELA and VSD in the Offspring

**FIGURE 5:**
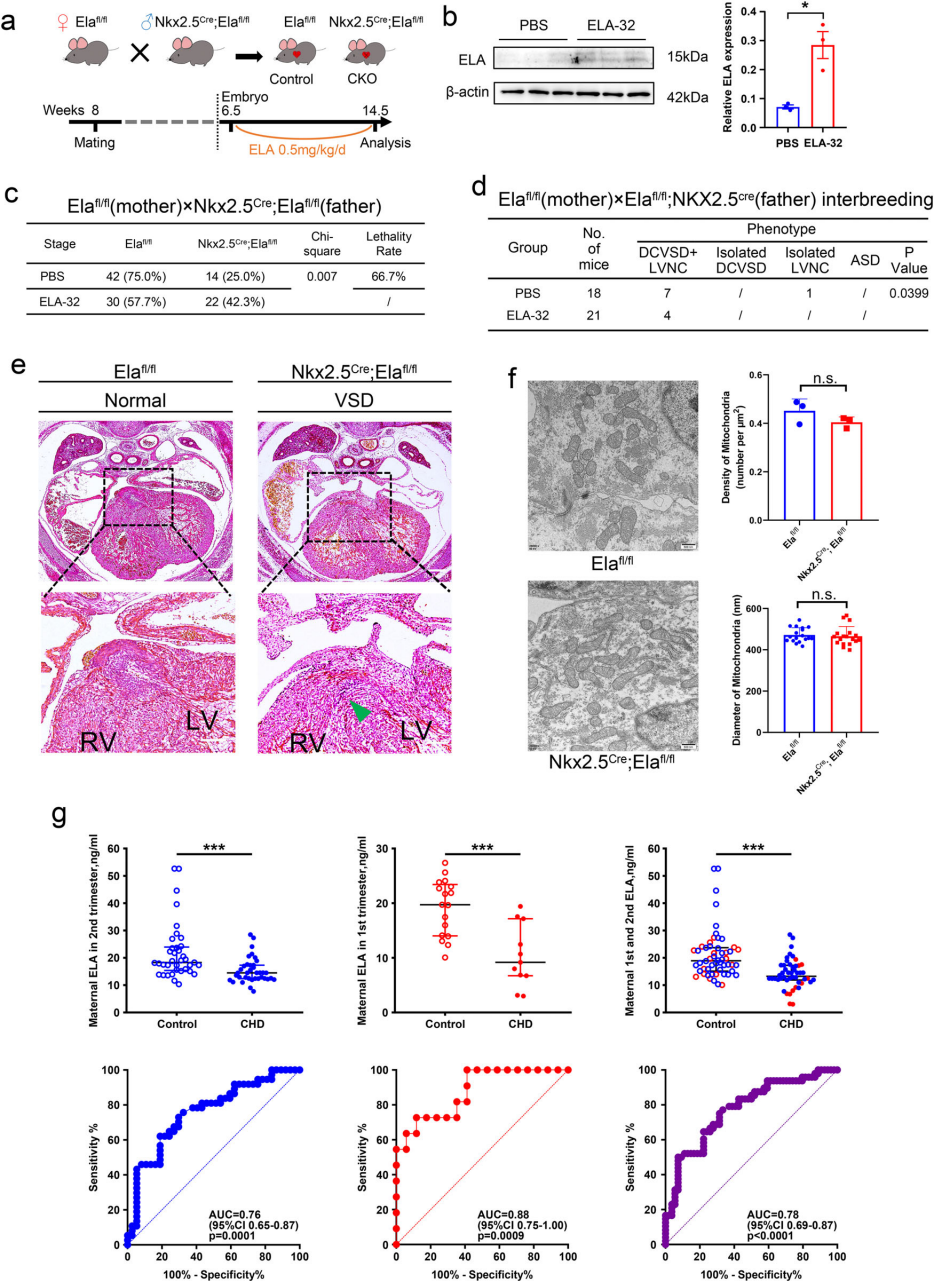
**ELA decreases the incidence of CHD in mice and ELA concentration is correlated with fetal CHD in human**. (a), Exogenous ELA administration by continuous subcutaneous infusion from E6.5 after mating. (b), The protein level of ELA in offspring heart after treatment pregnant mice with synthetic ELA peptide (n = 3). (c), Exogenous ELA administration reduced the mortality rate of offspring. (d), Phenotypic spectrum of ELA^Nkx^ mice after infusion of ELA. (e), CHD phenotypes in E14.5 mice embryos. Representative H&E staining results are shown in the right. (f), Transmission electron microscope of E11.5 heart of control and CKO embryos after exogenous ELA administration by continuous subcutaneous infusion through micropump (n = 3 and 18). Scale bars 2 um for 7000x, 500 nm for 2500x. (g), Upper left, maternal ELA concentration during first trimester significantly decreased in pregnancies with fetal CHD (n = 11) than matched controls (n = 17). Upper middle, maternal ELA concentration during second trimester significantly decreased in pregnancies with fetal CHD (n = 37) than matched controls (n = 37). Upper right, combined ELA concentration during both first and second trimesters (Control, n = 54; CHD, n = 48). Lower, receiver‐operator characteristic (ROC) curve of ELA concentration as a predictor of fetal CHD. The left is second‐trimester maternal ELA (AUC: 0.76), the middle is first trimester maternal ELA (AUC: 0.88), and the right is combined trimesters (AUC: 0.78). For all, ^*^ *p*<0.05, ^***^ *p*<0.001. Data are represented as mean ± SEM. For (b) and (f), Two‐tailed t test was applied. For (c) and (d), Chi‐square test was applied. For (g), upper panel, Two‐tailed t test was applied; lower panel, ROC curve analysis was applied.

Given that endogenous ELA is detectable by ELISA in mouse peripheral blood during pregnancy [16], we postulated whether administration of ELA during gestation could mitigate the incidence of cardiac anomalies in the offspring of ELA knockout embryos (Figure 5a). Moreover, subcutaneous administration of the ELA peptide to pregnant mice elevated ELA protein levels in offspring hearts (Figure 5b,c). This intervention significantly reduced embryo lethality (*p* < 0.01, Chi‐square test) and markedly decreased the incidence of cardiac anomalies in ELA‐deficient embryos (*p* < 0.05, Chi‐square test; Figure 5d). The administration of ELA peptide lowered the incidence of VSD in mouse (DCVSD, 7 out of 18 compared to 4 out of 21) (Figure 5d,e). Besides, no phenotypic manifestation of mitochondrial swelling was observed in ELA^Nkx^ embryonic hearts (Figure 5f). Together, these results highlighted the effectiveness of exogenous ELA on mitochondria in CMs.

In human studies, the concentration of ELA in plasma has been reported to correlate with various diseases [33, 34, 35, 36]. Yet, the potential association between maternal plasma ELA levels and CHD in offspring has not been previously investigated. Therefore, we conducted a nested case‐control study based on two prospective birth cohorts, the Shanghai Birth Cohort (SBC) and the Early Life Plan (ELP) [37], using plasma samples obtained from pregnant women during the first trimester (at gestational ages of 11–‐14 weeks) to explore this association. The study included a case group (N = 9) diagnosed with CHD through fetal echocardiography during pregnancy or transthoracic echocardiography after birth, and the matched control group (N = 17) were also from the two birth cohort platforms.

We observed that maternal plasma ELA levels were significantly lower in cases of fCHD during the first trimester (gestational weeks 11–‐13). Similarly, plasma ELA levels in the second trimester (gestational weeks 14–‐26) were also markedly reduced. Analysis of combined samples from both trimesters confirmed this trend (Figure 5g). The patients' basic characteristics are detailed in the supplementary materials (see Table S2). Receiver operating characteristic (ROC) curve analyses indicated that maternal ELA concentration was a reliable discriminator for fetal CHD, with area under the curve (AUC) values ranging from 0.76 to 0.88 (Figure 5g). Notably, the concentration of ELA in the first trimester demonstrated the highest discriminatory ability, achieving an AUC of 0.88 and a *p*‐value of less than 0.0009. This underscores the potential of maternal ELA levels as an effective biomarker for early detection of fCHD.

## 3 Discussion

This study integrates human tissue samples and mouse model systems to elucidate the critical role of ELA in both human embryonic heart development and the pathogenesis of CHD. Our results highlight the essential role of ELA in heart development and its link to the development of heart defects in human. Our data demonstrates that the mechanism by which ELA deficiency leads to abnormal embryonic heart development in mice. We also uncovered that ELA peptide is therapeutic for congenital heart disease and a potential biomarker for human CHD during pregnancy. These findings provide insight into the pathophysiology of CHD and underscore the importance of monitoring maternal ELA levels as a potential indicator of fetal heart health. Furthermore, our study contributes to the field not only by identifying the initial discovery of the pathway that regulating cardiac development, but also by translating it to human biology, clarifying its clinical relevance, and characterizing its mechanism.

Although previous researches have revealed that ELA is a key regulator of early embryogenesis, but mainly in animal models and in vitro cell culture systems [13, 18, 19], the link between ELA and human heart development remains largely unexplored. Our data first reveals that ELA may play a role during human heart development. Moreover, we discovered that the ELA expression is reduced in the cardiac tissue of human fetuses with CHD, providing the initial evidence of the link between ELA and human CHD. ELA is expressed in various human tissues and cell types [13, 36, 38, 39], yet it is notably absent in adult cardiomyocytes [36]. Interestingly, our study identified ELA expression in fetal CMs, suggesting a potentially unique role for ELA in heart development.

To further investigate the underlying mechanism of ELA on heart development, we generated an ELA‐lineage tracer mice, and the expression of ELA was detected during the heart development. Previous studies have shown that systemic knockout of ELA leads to cardiac anomalies during early development of mammalian embryos [16, 19] but with no specific subtype of cardiac defects due to early embryonic lethality around E10.5. Thus, the specific function of cardiac‐restricted ELA in the occurrence of CHD remains uncertain. Our cardiac‐specific ELA knockout mice revealed that ELA deficiency in the developing heart leads to DCVSD, ASD, and LVNC, illustrating a critical role of ELA in heart development. Our study revealed that the loss of ELA specifically in cardiomyocytes not only resulted in a 40% embryonic lethality rate but also caused abnormalities during cardiogenesis. This underscores the essential cardiomyocyte‐autonomous role of ELA in cardiac malformations, avoiding potential systemic crosstalk among different tissues due to conventional null alleles, which highlighted the specificity and importance of this study.

The heart has a massive demand for energy, relying on a vast pool of functional mitochondria to produce high‐energy phosphates [40, 41, 42]. Cardiomyocytes bear the intense metabolic and mechanical requirements of the heart, which is why mitochondria occupy most of the cell volume. Thus, mechanisms ensuring the health of cardiomyocyte mitochondria are critical for cardiac function and heart development. To prevent mitochondrial damage, cardiomyocytes possess a well‐coordinated quality control system that maintains the overall health of mitochondria by balancing three biological processes: mitochondrial biogenesis, dynamics, and autophagy [28, 29, 30]. Although energy depletion likely contribution to abnormal myocardial morphologies and cardiomyopathy [43, 44], understanding the mechanisms that sustain mitochondrial health still is a vital topic in researches of CHD.

Our study reveals the role of ELA in regulating mitochondrial function and its impact on a fundamental process of cardiac development: cardiomyocyte apoptosis. Apoptosis serves as a vital biological process during organogenesis [45, 46, 47]. Transgenic and knockout studies have collectively provided evidence that aberrant apoptosis of cardiomyocytes can lead to cardiac anomalies [46, 47]. In ELA knockout (ELA^Nkx^) embryos and ELA knockdown cells, the proportion of apoptotic cardiomyocytes appeared to be higher, concomitant with declined oxidative phosphorylation. Furthermore, this study demonstrate that the endogenous peptide ELA is essential for maintaining mitochondrial function in cardiomyocytes by activating the APJ‐AKT‐BCL2/BAX axis. A significant finding is that cardiomyocytes from ELA‐deficient mice show severe mitochondrial swelling, cristae collapse, and functional impairments. Remarkably, supplementing ELA in these mice leads to a reversal of mitochondrial swelling. Therefore, we conclude that ELA is both a necessary and sufficient factor for sustaining mitochondrial homeostasis in cardiomyocytes. Mechanistically, ELA deficiency disrupts mitochondrial function in cardiomyocytes by selectively altering dynamics (decreased MFN1, OPA1, p‐DRP1^S616^, TFAM, and PGC‐1α) and reducing membrane integrity, leading to mitochondrial permeability. These defects resulted in a severe bioenergetic deficit, characterized by loss of ATP synthase activity, reduced ATP production, and impaired OCR. Consequently, cells shifted toward glycolytic metabolism (increased ECAR) to compensate, highlighting a crucial role for ELA in maintaining cardiac metabolic homeostasis.

ELA acts as an endogenous secretory peptide that binds to the APJ receptor on the cell membrane, thereby activating the AKT‐BCL2/BAX signaling pathway. This activation is crucial for maintaining mitochondrial membrane integrity and provides protection against apoptosis by inhibiting the release of cytochrome c and other pro‐apoptotic factors. This highlights the role of ELA in not only supporting mitochondrial function but also in promoting cell survival. Our Single‐cell sequencing analysis revealed that cardiac‐specific knockout of ELA in mice altered not only the AKT‐BCL2/BAX signaling pathway but also other pathways (including MAPK signaling and Wnt signaling), suggesting potential crosstalk between these pathways. In future studies, we will use tissue‐specific knockouts and comprehensive phosphoproteomic analysis to further elucidate these complex mechanisms.

In studies focused on ischemia‐reperfusion injury, it has been observed that increased BAX expression exacerbates apoptosis in cardiomyocytes, adversely affecting cardiac function [48]. In contrast, therapeutic strategies that promote BCL2 overexpression effectively reduce apoptosis and enhance the tolerance of cardiomyocytes to ischemia‐reperfusion injury [49]. Our research underscores the significance of targeting the upstream regulator ELA in the BCL2/BAX pathway for the treatment of CHD. By prioritizing ELA over direct intervention on intracellular BCL2/BAX, we highlight the enhanced translational potential of ELA peptide therapy in improving cardiomyocyte function. This finding suggests that ELA not only plays a crucial role in maintaining mitochondrial health but also represents an important therapeutic target for CHD.

In this study, we also found that the administration of exogenous ELA peptide in pregnant mice dramatically mitigated the incidence of cardiac defects in ELA^Nkx^ embryos. This suggests that ELA, functioning as a circulating hormone, could traverse the placental barrier to reach the fetus, indicating that ELA peptide in maternal plasma may play a vital role in fetal heart development, serving as a promising therapeutic target for fetal CHD. Future researches should focus on understanding how ELA is transported across the placenta and factors contributing to decreased maternal ELA levels in pregnancies with fetal cardiac abnormalities. Investigating ELA as a potential therapeutic agent could enhance strategies for preventing CHD.

Furthermore, ELA could be a promising maternal biomarker for fCHD during early pregnancy. Presently, the examination of structural abnormalities in the fetal heart and cardiovascular system largely relies on imaging technology, with a time‐restricted window during gestation primarily concentrated in the second trimester [50, 51]. However, early screening and diagnosis of fCHD is crucial as it offers more time and opportunities for early counseling of families, reproductive choices, and referral to expert physicians in tertiary facilities [52, 53]. Our study addressed the critical role of ELA in cardiac development and explored its initial application in predicting fCHD in humans, especially with best efficacy during first trimester. However, the journey from discovery to clinical practice implementation of a biomarker is typically a complex and time‐consuming process. Rather than relying solely on the maternal level of ELA, a multidimensional and comprehensive risk assessment for fCHD during the first trimester is needed. A more profound understanding of the secretion, elimination, and biological effects of ELA, as well as external validation of its value in larger cohorts, could warrant further investigation.

## 4 Experimental Section

### 4.1 Methods

Human tissue and blood samples for this study were acquired for assessing the ELA protein level. All procedures received approval from the Ethics Committee of Xinhua Hospital Affiliated to Shanghai Jiao Tong University, School of Medicine (No. XHEC‐C‐2016‐016 and No. XHEC‐C‐2013‐001‐7, No. XHEC‐C‐2019‐083‐3 and No. XHEC‐F‐2017‐004‐2) and all experiments were performed in line with relevant guidelines and regulations. This investigation was designed and executed following the principles outlined in the Declaration of Helsinki.

All experimental studies involving animal subjects were carried out in strict adherence to the guidelines of the Institutional Animal Care and Use Committee of Xinhua Hospital, Shanghai Jiao Tong University School of Medicine, and were conducted following the National Institutes of Health Guidelines for the Care and Use of Laboratory Animals (No. XHEC‐F‐2024‐034‐1).

### 4.2 Study Structure and Participant Selection

This study was carried out at the Xinhua Hospital, which is affiliated with the Shanghai Jiaotong University. Comprehensive informed consent was obtained from all participants, who were made aware of their rights to voluntarily withdraw from the study at any stage.

In the second trimester phase of the investigation, a preliminary case‐control study was employed, with inclusion parameters for the pregnancies being: (1) a minimum maternal age of 20 years; (2) fetal CHD diagnosis confirmed by fetal echocardiography during the second trimester, or confirmed postnatally via trans‐thoracic echocardiography (TTE). Exclusion criteria encompassed pregnancies complicated by maternal hypertension, acute or chronic renal disorders [54], or periconceptional hyperglycemia. Controls were selected from the Shanghai Birth Cohort (SBC) [55], matched according to gestational age, enrollment timing, and blood pressure status. As per the modified classification by Schwedler et al. [56], mild CHD cases were defined as those that necessitated only postnatal follow up and exhibited no significant hemodynamic changes, such as minor ventricular septal defects, isolated atrial septal defects, etc. Sociodemographic data was collected at the time of initial enrollment.

The validation study, which utilized first‐trimester maternal plasma ELA concentration, was performed using samples from two prospective birth cohorts, namely the Shanghai Birth Cohort (SBC) and Early Life Plan. Inclusion criteria mirrored those of the preliminary study. Participants were triaged into three groups based on the severity of fetal CHD, with the control group also being matched from the SBC according to gestational age, enrollment timing, and blood pressure status. Both the second‐trimester case‐control study and the first‐trimester nested case‐control study obtained ethical approvals from the Ethics Committee of Xinhua Hospital Affiliated to Shanghai Jiao Tong University, School of Medicine (No. XHEC‐C‐2016‐016 and No. XHEC‐C‐2013‐001‐7).

### 4.3 Human Tissue Collection

Human tissue samples for this study were acquired post elective medical/surgical abortions at Xinhua Hospital Affiliated to Shanghai Jiao Tong University, School of Medicine, with written informed consent from the women. Post‐conceptional age was determined using clinical ultrasound and the stage‐dependent anatomical characteristics of embryos. All patients were over 18 years of age, of Han racial background, and without pre‐existing conditions that would pose risks to abortion. All procedures received approval from the Ethics Committee of Xinhua Hospital Affiliated to Shanghai Jiao Tong University, School of Medicine (No. XHEC‐C‐2019‐083‐3 and No. XHEC‐F‐2017‐004‐2), and all experiments were performed in line with relevant guidelines and regulations. For human embryonic tissues, 41 samples (n = 5 at CS11 and CS13; n = 3 at CS12, CS14, CS15; n = 4 at CS16) were analyzed by immunohistochemistry (IHC) and 61 (n = 7 at CS11; n = 9 at CS12; n = 12 at CS13 and CS14; n = 11 at CS15; n = 10 at CS16) by quantitative PCR (qPCR). For human fetal hearts, 3 samples were assessed via IHC, 6 (control, n = 3; case, n = 3) by immunofluorescence (IF), and 3 by qPCR.

### 4.4 Animals

*Ela* conditional knockout mice was generated with strategy shown in Figure S5d. *Ela* loxp (*Ela* ^flox/flox^, *Ela* ^f/f^) mouse was generated using the CRISPR‐Cas9‐mediated genome editing system on a C57BL/6 background by Shanghai Model Organisms Center, Inc., (Shanghai, China). The ELA‐DreER‐IRES‐GFP mice line was generated by professor Bin Zhou at institute CAS Center for Excellence in Molecular Cell Science, Shanghai Institute of Biochemistry and Cell Biology as previously described [57]. *Nkx2‐5* Cre mice were given away by professor Bin Zhang at institute for Developmental and Regenerative Cardiovascular Medicine, Xinhua Hospital Affiliated to Shanghai Jiao Tong University School of Medicine. Isoflurane gas (1%–3% at a flow rate of 0.25–1 L/min) was used to anaesthetize animals. Euthanasia was performed with anaesthesia with 5% isoflurane, followed by cervical dislocation. The primer used for genotype identification of the offspring are shown in Table S3. All procedures received approval from the Ethics Committee of Xinhua Hospital Affiliated to Shanghai Jiao Tong University, School of Medicine (No. XHEC‐F‐2024‐034‐1)

### 4.5 ELISA

Blood samples were collected at the first enrollment during first‐ or second‐ trimester. The venous blood samples were centrifuged for 10 min at 400 rpm immediately after collection. Serum was stored at −80°C. The ELA concentrations in EDTA anticoagulated plasma were then measured in duplicates by human ELA Elisa Kit (#S‐1508, Peninsula Laboratories International, Inc. USA).

### 4.6 Quantitative PCR

Total RNA was extracted from tissues using Tissue RNA Purification Kit PLUS (EZB, RN001‐plus). Reverse transcription of cDNA was performed using iScript gDNA Clear cDNA Synthesis kit (BIO‐RAD, 1725035) and was perfomed by quantitative RT‐PCR protocal using PowerUP SYBR Green Master Mix (ThermoFisher, A25741)on an Applied Biosystems 7500 system. The relative quantification of expression was determined using the 2^Δct×10^3^ method, and glyceraldehyde‐3‐phosphate dehydrogenase (GAPDH, human) was used as an internal control.

Total RNA for cells was extracted using TRIzol reagent (Invitrogen, 15596018), and reverse transcription of cDNA was performed with PrimeScript RT Master Mix (Takara, RR047A). Next, quantitative RT‐PCR was performed using SYBR Premix Ex Taq (Takara, RR420A) on an Applied Biosystems 7500 system. The relative quantification of expression was analyzed with the 2^‐ΔΔCt method. All primer sequences are listed in Table S4.

### 4.7 Imunofluorescence and Imunohistochemistry Assays

For immunofluorescence staning, tissues were fixed in 4% paraformaldehyde, embedded in OCT (Sakura), and sectioned at a thickness of 7–‐8 µm. Cryosections were dried at room temperature, then washed 3 times in 1 × PBS. Subsequently, the sections were blocked with blocking buffer (5% donkey serum and 0.1% Triton X‐100 in PBS) for 30 min at room temperature, followed by incubation with appropriately diluted primary antibodies at 4°C overnight. Primary antibodies were used as listed: ELA (Phoenix Pharmaceuticals, Inc, H‐007‐19; 1:100 dilution), TNNT (Abcam, ab8295; 1:100 dilution), TNNI3 (Abcam, ab56357; 1:100 dilution), Nkx2‐5 (Abcam, ab6673; 1:100 dilution), GFP (Nacalai, 04404–84; 1:200). Afterward, the sections were washed 3 times in PBS and incubated with Alexa fluorescence‐conjugated secondary antibodies at a dilution of 1:2000 (ThermoFisher Scientific) at room temperature for 30 min in dark. Cell nuclei were stained with 4, 6‐diamidino‐2‐phenylindole (DAPI) (Vector Laboratories). A Leica SP8 microscope was used for image analysis. For immunolocalization of ELA, paraffin sections were incubated with a primary rabbit anti‐ELA antibody (Phoenix, 1:500), followed by horseradish peroxidase conjugated secondary anti‐rabbit antibody (ThermoFisher Scientific; 1:2000) and DAB (Abcam). A Leica SP8 microscope was used for image analysis. Fluorescence intensity and H‐scores were quantified using ImageJ. The fluorescence intensity was calculated per section area. The H‐Score is calculated as: percentage of weak stained cells +2 × percentage of moderate stained cells +3 × percentage of strong stained cells, getting a score between 0 and 300.

### 4.8 TUNEL Assay and ROS Detection

Apoptosis and reactive oxygen species (ROS) were evaluated using TUNEL assay (Beyotime, C1090) and fluorescent probes (MitoSOX, Yeason, 40778ES50; Dihydroethidium, Beyotime, S0063), respectively, according to the manufacturer's. Following blocking and permeabilization, samples were incubated with TUNEL reaction mixture for 1 h at 37°C. For ROS detection, live cells were stained with 5 µmol/L MitoSOX for 10 min and Dihydroethidium for 30 min at 37°C. After PBS washing, all images were captured using a Leica DM2000 microscope and analyzed with ImageJ software.

### 4.9 Plasmid and Co‐Immunoprecipitation Assays

Full‐length Apela (NM_001297550) and Aplnr cDNAs (NM_005161) via PCR‐based cloning were inserted into the pcDNA3.1‐Flag and pCMV‐HA vector respectively, to generate expression constructs designated as ELA‐Flag and APJ‐HA. For co‐immunoprecipitation assays, HEK293 cells were co‐transfection with indicated plasmid. After 48 h, cells were lysed and the supernatants were incubated overnight at 4°C with anti‐HA and anti‐Flag antibody‐conjugated agarose beads (MCE, HY‐K0236 and HY‐K0237). Following extensive washing, bound proteins were eluted by boiling in SDS loading buffer and analyzed by Western blot.

### 4.10 Flow Cytometry Analysis

Primary cardiomyocytes were digested with trypsin after infecting with small interfering RNA for 24 h, resuspending to about 2^10^6^ cells. Cells were transferred to a flow cytometry tube, and stained with an appropriate amount of fluorescent dye‐labeled Annexin V and PI at room temperature for 15–‐20 min. After staining, the cells were washed, resuspend and evaluated using a BD FACSCanto II (BD Bioscience) as soon as possible (within 1 h). The data analysis was performed with FlowJo (Treestar Inc.).

### 4.11 Single‐Cell RNA Sequencing

E9.5, E11.5, and E14.5 embryonic hearts from groups of ELA^fl/fl^ mice line and Nkx2‐5^Cre^; ELA^fl/fl^ mice line were collected. The single cell suspensions were generated by treating individual hearts with 0.25% Trypsin (HyClone) for 20–‐30 min at 37°C with shaking. Digestion was filtered with a 40‐µm cell strainer (BD Falcon) to remove large clumps and quenched with DMEM with 10% fetal bovine serum. The cell pellets were inspected on the Countess II FL (ThermoFisher Scientific) and under a microscope for cell number, cell viability, and cell size. For E9.5 and E11.5 timepoints, we pooled hearts of the same genotype to acquire sufficient cells for loading onto the 10x instrument. For the E14.5 timepoint, we used individual hearts for each experiment. The scRNA seq libraries were generated using the v2 single cell reagent kit (10x Genomics), according to the manufacturer's instructions. The final libraries were sequenced using an Illumina Nextseq500.

### 4.12 Single‐Cell RNA‐Seq Data Processing

Single‐cell RNA sequencing data were processed using the Seurat package (v4.3.0.1) in R [58]. Raw unique molecular identifier (UMI) count matrices generated from the CellRanger 10X pipeline were imported and consolidated into a single Seurat object. Cells were excluded based on the following criteria: a percentage of mitochondrial counts exceeding 20%, a percentage of erythrocytic (Hbb or Hba) counts greater than 5%, or fewer than 200 unique features.

Gene counts for each cell were normalized to total expression, multiplied by 10 000, and then log‐transformed. The resulting normalized counts were mean‐centered and scaled according to their standard deviation. Principal component analysis (PCA) was utilized to achieve linear dimensional reduction of the scaled data. Subsequently, Uniform Manifold Approximation and Projection (UMAP) was performed [59] using the first 30 principal components to further condense the dimensionality of the dataset. Finally, hierarchical clustering was executed on the 30‐dimensional UMAP embeddings employing Seurat's default method (Louvain algorithm) at a resolution of 0.5, resulting in the identification of 30 cellular clusters. The determination of marker genes for each cluster was accomplished via the Wilcoxon rank‐sum test (Seurat's FindAllMarkers), and cell types were manually assigned based on the marker genes corresponding to each cluster.

### 4.13 Neonatal CM Isolation and Culture

Neonatal cardiomyocytes (NCM) were isolated from 1 to 2 days‐old C57BL/6 mouse hearts and Sprague‐Dawley rat hearts as previously described [60]. Briefly, the hearts were aseptically collected and placed in cold PBS, and then minced, digested, and incubated with prewarmed enzyme mix for 15 min at 37°C water bath with a magnetic stirring. DMEM supplemented with 10% FBS was added into enzyme mix to stop digestion. The digestive steps were repeated for 2–‐3 times to maximize yield. Following centrifugation at 100 × g for 5 min, the precipitated CMs were resuspended with culture medium (10% FBS/DMEM). The suspended cells were initially plated and incubated at 37°C for 1–‐2 h. Thereafter, the culture medium containing non‐adherent cells was collected to enrich for cardiomyocytes. These enriched cardiomyocytes were subsequently seeded into the 10 µg/ml laminin‐coated wells and cultured at 37°C in a 5% CO_2_ environment.

### 4.14 siRNA Transfection (6‐Well Plate)

The transfection complex is prepared by mixing 300 µL of Opti‐MEM medium (Gibco, serum‐free) with 5 µL of Lipofectamine RNAiMAX (Invitrogen) in a sterile tube, followed by gentle pipetting and incubation at room temperature for 5 min. 2 µL of siRNA (20 µm stock) is then added, mixed gently, and incubated for 15 min at room temperature. 2 mL of complete medium is added to each well of a 6‐well plate, after which the 300 µL siRNA‐lipid complex mixture is added dropwise, with gentle swirling to ensure even distribution. Before transfection, cells are washed once with PBS, and the 2.3 mL transfection medium mixture is added. Cells are incubated at 37°C, 5% CO_2_ for 4–6 h, after which the medium is replaced with fresh complete medium.

### 4.15 RNA Velocity Analysis

RNA velocity analysis was conducted by utilizing R package velocito.R v6.0 [61]. The expression of spliced and unspliced count matrices were generated separately for each sample. Then the resulting data were imported into AnnData format through Scanpy, based on the outputs from the velocyto directory. For the Seurat object with annotated cell types, only highly variable genes were retained prior to conversion to AnnData format. The spliced and expression matrices were subsequently merged into a single AnnData object using scvelo.utils.merge.

From six groups of control and CKO samples across three time points, 10 000 cells were randomly sampled from each group. The standard workflow of scvelo was executed with the mode parameter set to ‘dynamical.’ The velocity length and velocity confidence for each cell were calculated using the scvelo.tl.velocity_confidence function, and statistical testing was performed using a two‐tailed t‐test.

### 4.16 GO Analysis

For each cell type at every time point, differential expression analysis was performed using the FindMarkers function from Seurat, with ident.1 designated as CKO and ident.2 as Ctrl. Genes were classified as upregulated if they exhibited an average log2 fold change (avg_log2FC) greater than 0 and a *p*‐value less than 0.05, whereas downregulated genes were defined as those with an avg_log2FC less than 0 and a *p*‐value below 0.05. Subsequently, the clusterProfiler package [62] was utilized to identify enriched pathways associated with the differentially expressed genes.

### 4.17 Genomic DNA Extraction and PCR

Genomic DNA was got from mouse tails of postnatal mice or yolk sac for embryos. Tissues were incubated in lysis buffer (Vazyme) at 55°C for at least 45 min. Then the lysis buffer was boiled at 95°C for 5 min. Genomic DNA in supernatants was used for genomic PCR as reported previously [63]. Primer sequences were listed in Table S3.

### 4.18 Western Blot

Whole tissues and cells were lysed in RIPA buffer supplemented with protease inhibitor cocktail (Sigma–Aldrich) as described previously [64]. A total of 30 µg protein was electrophoresed and separated by 10% SDS‐PAGE and transferred to polyvinlidene difluoride membranes (ISEQ00010, Millipore). The membranes were blocked with 5% milk for 1 h at room temperature and incubated with indicated primary antibodies at 4°C overnight, then corresponding secondary antibodies for 2 h at room temperature. Blots were visualized using an enhanced chemiluminescence (Applygen). All antibodies for Western blot were: anti‐p‐AKT (Cell Signaling Technology, 4058S, 1:1000), anti‐t‐AKT^S473^(Cell Signaling Technology, 4685S, 1:1000), anti‐β‐Actin (ABclonal, AC004, 1:5000), anti‐BAX (Abways technology, CY5059, 1:1000), anti‐BCL‐2 (Abways technology, CY6717, 1:1000), anti‐PGC‐1α (Proteintech, 66369, 1:1000), anti‐TFAM (Santa Cruz, sc‐166965,1:100), anti‐DRP1 (Cell signaling technology, 8570S, 1:1000), anti‐MFN1 (Abcam, ab221661, 1:1000), anti‐OPA1 (Cell Signaling Technology, 80471, 1:1000), anti‐phospho‐DRP1^Ser637^ (Abcam, ab193216,1:1000), anti‐Parkin (Cell Signaling Technology, 2132, 1:1000), Anti‐Prohibitin (Abcam, ab75766, 1:1000), Anti‐LC3B (Cell Signaling Technology, 2775, 1:1000), Anti‐PINK (abcam, ab300623, 1:1000), Goat anti‐rabbit lgG (HCS) HRP conjugate (Abbkine, A25222, 1:2000), Goat anti‐rabbit lgG (LCS) HRP conjugate (Abbkine, A25022, 1:2000), Goat anti‐rabbit IgG (H+L) HRP conjugate (Abmart, M21002S,1:2000), Goat anti‐mouse IgG (H+L) HRP conjugate (Abmart, M21001, 1:2000).

### 4.19 Blue Native PAGE

To assess the native molecular mass and oligomeric status of mitochondrial protein complexes, Blue Native Polyacrylamide Gel Electrophoresis (BN‐PAGE) was performed as previously described [31, 65]. Briefly, mitochondria were isolated from mouse embryonic hearts and subsequently solubilized using a buffer containing 1% digitonin and 0.2% Coomassie G‐250 (Realtimes, DM1080). The resulting protein complexes were then resolved under native conditions on a 3.5% to 16% linear gradient gel (Beyotime, P0543S).

### 4.20 Transmission Electron Microscopy

The heart tissue of E11.5 was immersed in a solution of 2.5% glutaraldehyde and 2% paraformaldehyde, and incubated for 24 h at 4°C. After washing twice with PBS, the tissue was fixed with 1% osmium tetroxide and dehydrated by graded ethanol series (30%, 50%, 70%, 80%, 95% and 100%) at each step for about 10 min. The tissue was incubated with 100% propylene oxide and was immersed overnight in a 2:1 mixture of propylene oxide and embedding resin (epon 812), After being incubated for 6 h in the embedding resin at 37°C, the specimen was placed in capsules and heated at 60°C for 48 h. Then the specimen was sectioned and stained with uranyl acetate and lead citrate. Specimens were evaluated using a transmission electron microscope (HITACHI H‐7650). The quantification of mitochondrial structure in EM images was conducted according to well‐established methodologies described in previous studies [25, 26, 27].

### 4.21 Mitochondrial Permeability Transition Pore Assay

Mitochondrial permeability was measured using Mitochondrial Permeability Transition Pore Assay Kit (MPTP Kit) (Beyotime, C2009S) following the manufacturer's instructions. Specifically, cells were stained with Calcein AM (1X) and CoCl_2_ (1X) at 37°C for 30 min. Fluorescence intensity was measured using a fluorescence microplate reader.

### 4.22 Mitochondrial Membrane Potential Assay

Mitochondrial membrane potential was measured using JC‐1 (Beyotime, C2006) following the manufacturer's instructions. Specifically, cells were stained with JC‐1(1X) at 37°C for 20 min. Fluorescence intensity was measured using a fluorescence microplate reader.

### 4.23 Seahorse Assay

Oxygen consumption rate (OCR) and the extracellular acidification rate (ECAR) in real‐time were measured using the Seahorse XF Analyzer (Agilent Technologies) following established protocols for mitochondrial stress tests [31], with minor adaptations for embryonic cardiomyocytes. Briefly, neonate rat cardiomyocytes were seeded on XF96 microplates pre‐coated with Matrigel (Corning, 354277) at a density of 1 × 10^4^ cells/well and cultured overnight. OCR and ECAR were measured using the Mito‐stress test kit (Agilent, 103015–100) and glycolytic rate assay Kit (Agilent, cat#: 103020–100), respectively. Following equilibration in unbuffered assay medium, cells were sequentially exposed to metabolic modulators (glucose, oligomycin, FCCP, and rotenone/antimycin A) to evaluate mitochondrial respiration, glycolytic function, and metabolic flexibility. Data were normalized to total protein content and represent at least three independent experimental replicates.

### 4.24 Measurement of ATP Levels

Cellular ATP levels were detected using the Luminescent ATP Detection Assay Kit (Beyotime, S0026) according to manufacturer specifications. Briefly, adherent cells were lysed on ice with recommended lysis buffer (200 µL per well for 6‐well plates). The lysate was centrifuged at 12 000 × g for 5 min at 4°C to remove debris. The resulting supernatant was transferred to fresh tubes and combined with ATP working solution. Luminescent signals were immediately recorded using a luminometer, with absolute ATP concentrations determined through parallel standard curve analysis.

### 4.25 Statistical analysis

All statistical assessments were performed using an amalgamation of software applications, namely, Prism 8.0 software (Graphpad Software, Inc.), STATA Version 13.0 (Stata Corporation, College Station, TX, USA), SIMCA16.0.2 software (Sartorius Stedim Data Analytics AB, Umea, Sweden), and R version 6.0. Two‐tailed and Welch's t test were used for analysis comparison of mean values. Independent biological replicates are specified in each figure legend and data are represented with mean ± SEM unless otherwise noted. The diagnostic capacity of maternal ELA levels was assessed through the generation of Receiver Operating Characteristic (ROC) curves. *p*‐values less than 0.05 were deemed indicative of statistical significance.

## Author Contributions

J.W., Q.J.W., Z.K.X. and S.Z. contributed equally to this work. The study was conceptualized by K.S., A.F.Ch., J.Y.Zh., J.W., and Q.J.W., while the methodology was developed by Q.J.W., Z.K.X., S.Zh., J.J.Y., Y.Zh., Z.M., M.Y., and R.B.T. Investigations were conducted by J.W., H.L.W., and T.Y., with resources provided by J.Z., Y.Q.H., L.W., B. Zhang, B. Zhou, and S.C. The original draft was prepared by J.W., Q.J.W., Z.K.X., and L.F.T., and subsequently reviewed and edited by W.Zh., J.X.Y., A.F.Ch., J.Y.Zh., B. Zhou, and B. Zhang. Funding was acquired by A.F.Ch., K.S., and J.W., and project administration was managed by K.S. and A.F.Ch. The study was conducted under the supervision of J.Y.Zh. and K.S.

## Conflicts of Interest

The authors declare no conflicts of interest.

## Supporting information




**Supporting File**: advs75030‐sup‐0001‐SuppMat.docx.

## Data Availability

The data that support the findings of this study are available from the corresponding author upon reasonable request.
