# Supplementary material for: ELABELA Targets Mitochondria to Modulate Heart Development
Source: Adv Sci (Weinh). 2026 Apr 7;13(33):e06525. doi: 10.1002/advs.202506525 (PMC13271604; doi:10.1002/advs.202506525)
Supplement: Supplementary file 1 — Supporting File: advs75030‐sup‐0001‐SuppMat.docx. [file ADVS-13-e06525-s001.docx]

Supplementary data for

**ELABELA targets mitochondria to modulate heart development**

**Authors:** Jian Wang^1,2,3,11^, Qingjie Wang^1,2,3,11^, Zhikang Xu^1,2,3,11^, Shuang Zhou^1,11^, Yue Zhou^1,2^, Junjie Yang^1,2,3^, Lingfeng Tong^1,2,3^, Zhuo Meng^1^, Mei Yang^2,4^, Wen Zhao^1,2^, Tie Yang^1,2,3^, Hualin Wang^1^, Jun Zhang^5^, Rubin Tan^6^, Lei Wang^7^, Yuqiang Huang^8^, Bin Zhou^9^, Sun Chen^1,3^, Bing Zhang^2^, Jinxiang Yuan^10^*, Jianyuan Zhao^2,5^*, Alex F. Chen^2^*, Kun Sun^1,2,3,12^*

**Affiliations:**

^1^Department of Pediatric Cardiology, Xinhua Hospital Affiliated to Shanghai Jiaotong University School of Medicine, Shanghai, 200092, China.

^2^Institute for Developmental and Regenerative Cardiovascular Medicine, Xinhua Hospital Affiliated to Shanghai Jiaotong University School of Medicine, Shanghai, 200092, China.

^3^Engineering Research Center of Medical Devices for Congenital Heart Disease, Ministry of Education, Xinhua Hospital, Shanghai Jiao Tong University School of Medicine; Shanghai, 200092, China.

^4^Department of Cardiology, Xinhua Hospital Affiliated to Shanghai Jiaotong University School of Medicine, Shanghai, 200092, China.

^5^Ministry of Education-Shanghai Key Laboratory of Children’s Environmental Health, Xinhua Hospital, Shanghai Jiao Tong University School of Medicine, Shanghai, 200092, China.

^6^Department of Physiology, Basic medical school, Southwest Medical University, Luzhou, Sichuan, 646099,China.

^7^Diagnosis and Treatment Center for In Utero Pediatric Diseases, Xinhua Hospital Affiliated to Shanghai Jiao Tong University School of Medicine, Shanghai, China.

^8^Linyi Maternal and Child Health Care Hospital, Linyi, Shangdong Provice, 276000, China.

^9^New Cornerstone Science Laboratory, State Key Laboratory of Cell Biology, Shanghai Institute of Biochemistry and Cell Biology, Center for Excellence in Molecular Cell Science, Chinese Academy of Sciences, University of Chinese Academy of Sciences, Shanghai, China.

^10^Lin He's Academician Workstation of New Medicine and Clinical Translation, Jining Medical University, Jining, Shandong, 272067, China.

^11^These authors contributed equally

^12^Lead contact

*Correspondence: yuanjinxiang18@outlook.com (J.X.Y.); zhaojy@vip.163.com (J.Y.Zh); chenfengyuan@xinhuamed.mail.com.cn (A.F. Ch); sunkun@xinhuamed.com.cn (K.S.)

**Figure S1 Generation and characterization of Ela-DreER and *ELA* knockout mouse lines. a**, qPCR of the relative mRNA level of ELA in mouse embryonic hearts. **b**, Schematic diagram showing the knock in strategy of ELA-Dre by homologous recombination. **c**, Immunostaining for GFP and Nkx2.5 in ELA-Dre E8.5 embryos. Lower panels show higher-magnification views of the boxed regions above. The expression of GFP represents that of ELA. Scale bars, 20um. **d**, Targeting of the ELA locus. Exon2 of murine ELA was flanked with loxp sites and excised with cre recombinase to generate the ELA knockout allele. **e**, Expression of ELA by qRT-PCR and the semi-quantitative PCR of ELA locus from gDNA. Data are represented as mean ± SEM. **f**, Schematic diagram illustrating the experimental strategies. *****P*<0.001. For (e), data are represented as mean ± SEM; Two-tailed t test was applied.

**Figure S2 Mouse cardiac-specific deletion of ELA reduces survival of embryos due to pericardial edema.** **a**, Penetrance of affected ELA conditional knock out (CKO) phenotypes at E9.5 and E12.5. **b**, Affected ELA CKO embryos have pericardial edema and intrauterine growth restricted. For (a), Chi-square test was applied.

**Figure S3 Single cell sequencing analysis of mouse embryonic heart tissue.** **a**, Left, Uniform manifold approximation and projection (UMAP) plot shows the topological structures of E9.5, E11.5 and E14.5. Right, UMAP plot shows unsupervised clustering of 30 groups from integrated cell data of mouse embryonic heart. Each cluster is color-coded. **b**, Dot plot illustrating the expression of marker genes for each cell population. c, The RNA velocity plots of control and CKO samples at E9.5, E11.5 and E14.5. CM, cardiomyocytes; EndoC, endocardial cells; Epi, epithelial cells; EpiC, epicardial cells; Mes, mesenchymal cells; MΦ, macrophages; FB, Fibroblast cells. Ctrl, Control; CKO, Conditional knock out.

Figure S4 ELA Deficiency Alters Mitochondrial Dynamics Protein Expression and Impairs Function. a-e, Quantification of (a) width of mitochondria, (b) lenth of mitochondrial, (c) lipid droplet area, (d) diameter of lipid droplet, (e) lipid droplet perimeter in control and ELA CKO embryonic hearts (n==36 for a and b, n=8-12 for c and e, n=8-19 for d). **f**, mRNA expression levels of ELA were measured by qRT-PCR 48 hours after transfection with 20nM of siRNA in neonatal mouse cardiomyocytes and neonatal rat cardiomyocytes (n=5 for left, n=3 for right). g, Total ROS analysis and MitoROS analysis of NCMs. h, mRNA expression levels of APJ were measured by qRT-PCR 48 hours after transfection with 20nM of siRNA (n=3). β-actin was used as an internal control. i, extracellular acidification rate (ECAR), which were normalized to total protein content. For all, **P*<0.05, ***P*<0.01, **** *P*<0.0001. Data are represented as mean ± SEM. Two-tailed t test was applied for (a-j).

**Figure S5.** **Analysis of mitochondrial dynamics, cristae architecture, and apelin protein in control and ELA CKO embryonic hearts by Western blot. a,** Blue native page analysis for heart tissue of control and CKO embryos (n=3 per group). **b,** Western-blot and semiquantitative analysis of key proteins of mitochondrial fission/fusion, biogenesis and pathways of mitophagy in control and ELA CKO embryonic hearts (n=4-6 per group**)**. **c,** Western-blot of apelin in control and ELA CKO embryonic hearts (n=6 per group).  For all, **P*<0.05, ***P*<0.01. Data are represented as mean ± SEM. Two-tailed t test was applied**.**

Table S1. Cell type-specific marker genes used in scRNA-seq data analysis.

| Cell Types | Markers |
| --- | --- |
| Cardiomyocytes | Tnnt2, Myh6, Actn2, Pln, Nkx2.5 |
| Fibroblasts | Col1a1, Postn, Col5a1, Tcf21 |
| Endothelial cells | Pecam1, Cdh5, Egfl7 |
| Epicardial cells | WT1, Upk3b, Tbx18, Upk1b |
| Smooth muscle cells | Acta2, Tagln |
| Macrophages | Apoe, Cd68, Cd86, C1qa, C1qb |
| Epithelial cells | Epcam, Krt18 |

Table S2. Demographic and clinical characteristics of the study population

| **tient characteristics** | **1^st^ trimester** | | | **2^nd^ trimster** | | |
| --- | --- | --- | --- | --- | --- | --- |
|  | **CON**  **(n=17)** | **CHD**  **(n=11)** | **P-value** | **CON**  **(n=37)** | **CHD**  **(n=37)** | **P-value** |
| Gestational age at sampling (w) | 14.75±1.75 | 14.74±1.86 | 0.867 | 24.45±0.82 | 26.29±2.14 | 0.000 |
| Maternal age at pregnancy (y) | 29.36±2.53 | 28.73±2.10 | 0.513 | 27.56±2.61 | 30.27±4.58 | 0.004 |
| Height (cm) | 162.93±6.74 | 161.64±4.11 | 0.582 | 161.65±4.57 | 161.72±4.77 | 0.947 |
| Weight before pregnancy (kg) | 57.26±10.61 | 56.09±7.78 | 0.762 | 55.66±7.69 | 54.46±7.13 | 0.501 |
| Weight (kg) | 60.36±14.44 | 58.8±9.52 | 0.761 | 58.31±7.01 | 62.12±7.86 | 0.039 |
| Maternal BMI before pregnancy (kg/m^2^) | 21.44±2.66 | 21.43±2.52 | 0.991 | 21.29±2.73 | 20.79±2.34 | 0.417 |
| BMI (kg/m^2^) | 22.52±3.51 | 22.44±3.06 | 0.957 | 22.34±2.72 | 23.74±2.56 | 0.032 |
| Race |  |  | - |  |  | 0.521 |
| Han | 11 (100%) | 11 (100%) |  | 34(46.2%) | 36 (97.3%) |  |
| Others | 0 (0%) | 0 (0%) |  | 0 (0%) | 1 (2.7%) |  |
| Hypertension |  |  | - |  |  | 0.545 |
| Yes | 0 (0%) | 0 (0%) |  | 30(100%) | 35 (97.2%) |  |
| No | 13 (100%) | 10 (100%) |  | 0 (0%) | 1 (2.8%) |  |
| Educational level |  |  | 0.792 |  |  |  |
| High school or less | 2 (14.3%) | 2 (18.2%) |  | 2 (5.9%) | 4 (11.4%) | 0.351 |
| Bachelor or above | 12 (85.7%) | 9 (81.8%) |  | 32 (94.1%) | 31 (88.6%) |  |
| Smoking during early pregnancy |  |  | - |  |  | - |
| Yes | 0 (0%) | 0 (0%) |  | 0 (0%) | 0 (0%) |  |
| No | 14 (100%) | 11 (100%) |  | 34 (100%) | 37 (100%) |  |
| Alcohol during early pregnancy |  |  | 0.366 |  |  | 0.021 |
| Yes | 11 (7.1%) | 0 (0%) |  | 5 (15.2%) | 0 (0%) |  |
| No | 13 (92.9%) | 11 (100%) |  | 28 (84.8%) | 36 (100%) |  |

Table S3. Primers sequences used for mouse genotyping

| Mice | Forward (5’-3’) | Reverse (5’-3’) |
| --- | --- | --- |
| ELA DreER | CATCTCCCACACCAAGACCATC | CACCCAGGCATCGCTAAAAATC |
| GFP | ACTTCTTCAAGTCCGCCATGC | TGATGCCGTTCTTCTGCTTGTC |
| ELA^fl/fl^ | TAAGCAGATTATCAAGAGAGCA | ACCCTTCAACTTTATGTGTCA |
| Nkx2-5 Cre | TTACGGCGCTAAGGATGACT | GTGTGGAATCCGTCGAAAGT |

Table S4. Primers sequences used for Real-Time PCR analysis

| Gene | Forward (5’-3’) | Reverse (5’-3’) |
| --- | --- | --- |
| h-ELA | GAAGAAGAAGAGGAGTGAAGGA | CCATTCCAGGTGCTTTCAAAT |
| h-GAPDH | TTGCCCTCAACGACCACTTT | TGGTCCAGGGGTCTTACTCC |
| r-β-actin | CTTCCTGGGTATGGAATCCT | TCTTTACGGATGTCAACGTC |
| r-ELA | CAGACACAACTGCTTTCGCC | AGGAGATCAGTGACTTTCTCATGG |
| m-ELA | CAGAAACCAGTTAACTTTCCCAGG | TGGGAAGGGCACTCGAGAAT |
| m- GAPDH | CAGGTTGTCTCCTGCGACTT | GGCCTCTCTTGCTCAGTGTC |
| m-m-mtNd1 | GGCCCTAACATTGTTGGTCC | TGGGTGTGGTATTGGTAGGG |
| m-mtNd2 | TCCTGTTAGTGGTGGAAGGC | CCTTACAACCCATCCCTCACT |
| m-mtNd3 | CTAGTTGCATTCTGACTCCCC | TGCTCATGGTAGTGGAAGTAGA |
| m-mtNd4 | CTAATAATCGCACATGGCCTC | CGTAGTTGGAGTTTGCTAGG |
| m-mtNd4l | TCTTCAACCTCACCATAGCCT | AGATGGTGATGGGGATTGGT |
| m-mtNd5 | CATCCTTCTCAACTTTACTGGG | TTTATGGGTGTAATGCGGT |
| m-mtNd6 | GGGATGTTGGTTGTGTTTGGA | CTACCCCAATCCCTCCTTCC |
| mNdufa12 | TCCTGAGTGGCACCGC | CTCTGGAGTGGCAGACACATT |
| m-mtCo1 | ACACAACTTTCTTTGATCCCG | AGAATCAGAACAGATGCTGG |
| m-mtCo2 | ATAATCCCAACAAACGACCT | CTCGGTTATCAACTTCTAGCA |
| m-mtCo3 | GGTATAATTCTATTCATCGTCTCGG | AGAACGCTCAGAAGAATCCT |
| m-mtCytb | CCATTCTACGCTCAATCCCCA | AGGCTTCGTTGCTTTGAGGTA |
| m-Atp6 | CCTTCAATCCTATTCCCATCC | GTTGGAAAGAATGGAGACGG |
| m-Bax | AGGCCTCCTCTCCTACTTCG | CCTTTCCCCTTCCCCCATTC |
| m-Bcl2 | AGCATGCGACCTCTGTTTGA | GCCACACGTTTCTTGGCAAT |

Table S5. Small interfering RNA sequences for knock down of *Ela* in primary CMs

| Name | Forward (5’-3’) | Reverse (5’-3’) |
| --- | --- | --- |
| Negative Control | UUCUCCGAACGUGUCACGUTT | ACGUGACACGUUCGGAGAATT |
| mEla | CUAUACAGACACAACUGCUTT | AGCAGUUGUGUCUGUAUAGTT |
| rEla | GAUCCAUAGUGGUGGGUUUUU | AAACCCACCACUAUGGAUCUU |
| rAPJ | CCACUGACAUCCCGGAAAAUU | UUUUCCGGGAUGUCAGUGGUU |
